# Understanding a Minority Group's (Roma) Experiences of Access and Quality in Maternity Services

**DOI:** 10.1111/hex.70389

**Published:** 2025-08-18

**Authors:** Jaspreet Kaur Dullat, Thérèse McDonnell, Louise Hendrick, Léan McMahon, Gemma Moore, Emily Murphy, Carmen Nae, Danut Nae, Marianna Prontera, Eilish McAuliffe

**Affiliations:** ^1^ Centre for Interdisciplinary Research, Education and Innovation in Health Systems (IRIS), UCD School of Nursing Midwifery and Health Systems Dublin Ireland; ^2^ National Quality & Patient Safety Health Service Executive (HSE) Ireland; ^3^ National Women and Infants Health Programme (NWIHP) Health Service Executive (HSE) Ireland; ^4^ Roma Programme Cairde Dublin Ireland

**Keywords:** experience, maternity services, minority, quality, Roma

## Abstract

**Background:**

Maternal health is a fundamental human right, ensuring that all women have access to non‐discriminatory, equitable and high‐quality maternal and reproductive healthcare. Minority groups and migrant populations face significant challenges in accessing maternity services. The Roma are Europe's largest and most marginalised ethnic group. Ireland has a sizable population of Roma migrants, and there is limited information on their experiences of maternal care in Ireland. Their participation in research and user panels is challenging as many lack proficiency in English and tend to have low levels of health literacy. By engaging Roma women in a unique co‐design education and research process, this study aims to identify barriers to accessing care faced by this minority community and understand their experiences of maternity services to enhance equity, accessibility and cultural responsiveness in maternal healthcare.

**Methods:**

This study employed a co‐design approach in partnership with Cairde, a community health organisation, to engage Roma women in exploring their experiences while engaging with maternity services. Eight Roma women participated in four online workshops and completed a questionnaire to share their experiences of pregnancy, childbirth and postnatal care. Workshops incorporated information components, focus groups and Romani translation and interpretation to ensure accessibility. Ethical considerations were maintained, and inductive thematic analysis was conducted using NVivo 20.

**Results:**

The study involved eight Roma women aged 21–39, many of whom had their first pregnancy between the ages of 14 and 19 and experienced homelessness after the birth of their first child in Ireland. Proficiency in English was limited; five women reported needing a translator while engaging with maternity services. Key patterns identified in Roma women's experiences of maternity care included language barriers, inadequate translation and interpretation support, and systemic discrimination, which leads to delays in antenatal care and heightened emotional distress. Additionally, systemic discrimination manifested through negative interactions with hospital security and administrative staff fostered mistrust and disengagement from healthcare services. However, despite these barriers, many Roma women expressed a preference for Irish maternity care over Romanian services, citing respectful treatment from healthcare professionals, shorter hospital stays, and the provision of essential postnatal supplies.

**Conclusions:**

This study highlights the significant barriers Roma women face in accessing maternity care in Ireland, including language barriers, financial constraints, cultural factors and discrimination. Limited English language proficiency, lack of translators and interpreters, and unfamiliarity with antenatal services often led to delayed or inadequate care, increasing health risks. Despite appreciating respectful treatment from healthcare providers, systemic challenges such as discriminatory practices and low health literacy persist. To address these disparities, it is essential to implement culturally tailored interventions, including the active recruitment and inclusion of Roma women across all levels of the healthcare system. This not only enhances the accessibility and quality of translation and support services but also fosters greater inclusivity, representation and culturally competent care. Future research should focus on expanding representation and designing and evaluating community‐based health initiatives.

**Patient or Public Contribution:**

This study collaboration between academic researchers, staff from the Health Service Executive (HSE), and Cairde, a community health development organisation, aimed to involve all stakeholders, including members of the Roma Community, in the co‐design approach that allowed the capture of the experiences and perspectives of Roma women on engaging with maternity services while giving birth in Ireland. Staff from Cairde, including members of the Roma Community, co‐designed a series of workshops and a questionnaire and provided support on the set‐up and delivery of the workshops and questionnaire. This collaboration resulted in the successful delivery of focus groups, informational sessions and a questionnaire, with full participation from the eight participants. Two of the eight Roma women who participated in the workshops were also involved in the co‐design process. Staff from Cairde, including members of the Roma Community, also contributed to the interpretation of findings and drafting of the papers. Partnering with Cairde afforded privileged access to the Roma Community, allowing the research team to engage with this seldom‐heard group. The many barriers to this engagement were addressed through partnering with an organisation with the trust of the Roma community and with the capacity to participate.

## 1 Background

Maternal health is a fundamental human right, aimed at ensuring that all women have access to non‐discriminatory, equitable and quality maternal and reproductive healthcare. To ensure women's maternal healthcare rights, health facilities, goods and services should meet standards of availability, accessibility, acceptability and quality [1]. However, international research highlights significant barriers faced by Roma women across Europe in accessing these essential services [2, 3].

The Roma are Europe's largest and most marginalised ethnic group, facing extreme poverty, racial discrimination, undermining of their cultural heritage, and exclusion from main society [4, 5]. Van Belle and Hughes (2022) highlighted that Roma women experience lower prenatal care engagement than other ethnic groups and increased maternal and neonatal complications [6, 7]. Socio‐economic disadvantages such as poverty, low education, inadequate nutrition, housing, and accommodation instability contribute further to poor obstetric outcomes [8, 9].

Roma women tend to marry and have children at a younger age compared to the general population [10]. A higher number of teenage pregnancies, high birth rates (average of 3.54 surviving children per woman), combined with low utilisation of antenatal services, put both mothers and infants at a higher risk of complications such as preterm birth, low birth weight and postpartum infection [11, 12]. Language barriers and poor healthcare literacy discourage Roma women from seeking timely maternity care [13, 14]. Additionally, systemic discrimination, including negative interactions with healthcare professionals, racial profiling and lack of culturally appropriate care, deters Roma women from accessing maternal health services, further widening existing healthcare disparities [2, 13].

In Ireland, the estimated Roma population is 16,059 (Census 2022) [15]. Studies indicate that 70% of Roma have lived in Ireland for over 5 years, with 63.3% of children in Roma households born in Ireland [16]. Despite this, Roma women continue to experience significant barriers to maternal healthcare access [17].

A report by Pavee Point Traveller and Roma Centre identified poverty, lack of social protection, absence of culturally appropriate information, and inadequate translation and interpretation services in Ireland as key concerns [17, 18]. Findings from the National Roma Needs Assessment (RNA) 2018 further emphasise the severity of these issues [17]:

- 24% of Roma women had their first contact with healthcare services during childbirth.
- 37.1% of Roma women reported insufficient postnatal supplies for their newborns, with many living in homes without heat, food or necessities.

70.5% of Roma individuals reported experiencing discrimination when attempting to access healthcare services. Maternity care in Ireland is provided free under the Maternity and Infant Scheme. The scheme offers combined care—by a family doctor (also called a general practitioner [GP]) in the community, and a hospital obstetrician or midwife, qualified to provide care for women and babies. During pregnancy, the initial examination is typically conducted by a GP, preferably before 12 weeks, to confirm the pregnancy and to register the mother on the maternity scheme. Further five examinations during the pregnancy are alternated with visits to the maternity unit/hospital. GP visits typically include monitoring progress and conducting routine examinations like weight and blood pressure monitoring or urine tests, whereas detailed examinations like ultrasound scans are carried out at the hospital level [19].

Many Roma women do not engage with maternity services early in pregnancy because they are unaware that the service is free and fear that care will be costly, compounded by fear of discrimination and communication barriers [18]. Discrimination and lack of culturally appropriate care negatively impact their experiences during pregnancy and childbirth [7]. Issues with translation and interpreter services in maternity units are particularly concerning and vary between hospitals, impacting Roma women's ability to attend appointments and communicate with healthcare providers [17].

Despite these challenges, there is a significant gap in research on effective interventions to improve maternity health services for Roma women [1]. To ensure that Roma women's voices inform healthcare service improvement, it is essential to actively involve them in shaping national maternity quality and safety services [20]. A more inclusive approach—one that prioritises accessibility, trust‐building and cultural appropriateness has the potential to significantly improve Roma maternal health outcomes [21].

The primary aim of this study was to capture the perceptions and experiences of Roma women on quality and safety in maternity services and to identify barriers to quality care. This study is part of a larger project developing quality and safety measures for maternity and neonatal services in Ireland. Recognising the importance of including marginalised groups in the development process, a targeted engagement strategy was co‐designed with Cairde (a community health organisation supporting marginalised populations) [22] to involve Roma women in shaping maternity care quality and safety measures. This approach ensures that their lived experiences can enhance service provision.

## 2 Methodology

This study utilised a co‐design approach outlined by Chauhan et al. (2021) to engage with Roma women in evaluating the quality and safety of maternity services [23]. To ensure meaningful participation, the research team collaborated with Cairde and recruited eight women originally from Romania who had given birth in Irish hospitals within the past five years.

A structured yet flexible methodology was adopted, including four online workshops and an online questionnaire co‐designed with Cairde's staff. Each workshop incorporated an educational component delivered by a Health Service Executive (HSE) Public Health doctor, followed by focus group discussions around antenatal care, care during labour and birth, and postnatal and neonatal care to capture the experiences of Roma women (Table 1). Language barriers were addressed through Romani translation provided by Cairde's staff, and literacy was addressed using graphics rather than words in education materials. Focus group discussions were conducted in small breakout rooms to encourage active participation using guided questions (Appendix A).

**Table 1:** Workshops: Educational component and focus group discussions.

Workshop	Educational component	Topic for focus group discussions
Workshop 1	You and your Pregnancy	Accessing antenatal care and barriers; language issues
Workshop 2	Having your Baby	Accessing healthcare during pregnancy and labour
Workshop 3	You and your Baby	Postnatal care, mental health and group session on designing a maternity service to meet the needs of Roma women and their babies
Workshop 4	—	

Ethical considerations, including informed consent and confidentiality, were upheld. To ensure accessibility and cultural sensitivity, participants received participant information leaflets (PILs) and consent forms (Appendices B and C), co‐designed with Cairde. Translation and interpretation support were provided by Cairde's staff to help participants fully understand the study process. Findings from the workshops were further validated through debriefing sessions. Upon completing the workshops, participants filled out an online questionnaire (Appendix D) to capture their background data and additional insights into their experience of maternity services using multiple‐choice and open‐ended questions. A descriptive analysis summarised key trends and highlighted patterns in healthcare utilisation and challenges faced by Roma women. The detailed methodology for this co‐design approach is outlined in a companion paper, which provides a comprehensive account of the engagement process [24].

## 3 TA Approach

We employed thematic analysis (TA) as outlined by Braun and Clarke in 2006 [25] to systematically develop themes to understand the lived experiences of Roma women within maternity services. Using an inductive approach, the transcribed interview data was collectively coded and analysed by members of the academic research team (J.D. and E.M.) using NVivo 20 software to identify key themes and sub‐themes.

## 4 Results

### 4.1 Sample Characteristics

Eight Roma women participated in focus groups to explore their experiences with maternity and neonatal services in Ireland (Table 2). Participants ranged in age from 21 to 39 years. The number of children per participant varied, with five women having more than five children. Three women were pregnant at the time of the study, and all had at least three children born in Ireland. Seven women reported becoming pregnant before the age of 18 years, and five women had experienced homelessness after the birth of their first child in Ireland.

**Table 2:** Sample characteristics.

Sample characteristics	No. of participants
Age (in years)	
20–25	1 (12.5%)
26–30	2 (25%)
31–35	3 (37.5%)
Above 35	2 (25%)
Number of years lived in Ireland	
Less than 5 years	3 (37.5%)
5–15 years	4 (50%)
More than 15 years	1 (12.5%)
No. of children	
1–2	1 (12.5%)
3–4	2 (25%)
5 and more	5 (62.5%)
No. of children born in Ireland	
Less than 3	5 (62.5%)
3 and more	3 (37.5%)
Age at the time of first pregnancy	
Less than 15 years	1 (12.5%)
15–18 years	6 (75%)
More than 18 years	1 (12.5%)
Age of the youngest child now	
< 2 years	2 (25%)
2–5 years	4 (50%)
> 5 years	2 (25%)
Are you pregnant now	
Yes	3 (37.5%)
No	5 (62.5%)
Place of birth	
Romania	7 (87.5%)
Other	1 (12.5%)
Experienced homelessness since the birth of your first child	
Yes	5 (62.5%)
No	3 (37.5%)
Experienced homelessness in Ireland	
Yes	5 (62.5%)
No	3 (37.5%)
Level of English (understanding and speaking)	
Very well	2 (25%)
Well	3 (37.5%)
Not well or not at all	3 (37.5%)
Level of English (reading)	
Very well	Nil
Well	3 (37.5%)
Not well or not at all	5 (62.5%)
Needed an interpreter at the hospital	
Yes	5 (62.5%)
No	3 (37.5%)
When needed an interpreter, how often it was provided by the hospital	
Always when asked	1 (20%)
Never on asking	3 (60%)
Never asked	1 (20%)
During the first pregnancy, were you aware that maternity care is free in Ireland	
Yes	4 (50%)
No	4 (50%)
Awareness regarding the number of recommended antenatal appointments	
Yes	1 (12.5%)
No	5 (62.5%)
Knew about some but not all	2 (25%)
At the time of first pregnancy, you attended a family doctor (GP) and/or hospital for pregnancy check‐ups/appointments before the birth of your baby in Ireland	
Yes, all or most of my appointments	3 (37.5%)
Yes, one or two	3 (37.5%)
No	2 (25%)
Attend GP/hospital for a 6‐week postnatal check‐up	
Yes	7 (87.5%)
No	1 (12.5%)
Receive a visit at home from the public health nurse	
Yes	8 (100%)
No	Nil
Method of feeding newborn	
Only breastfed	1 (12.5%)
Only bottle‐fed	4 (50%)
Both breastfed and bottle‐fed	3 (37.5%)
Had everything needed to care for the baby after the birth of the first baby in Ireland	
Yes, I didn't need any help	4 (50%)
Yes (hospital/charity/friends helped)	2 (25%)
No, I did not have what I needed but I managed	1 (12.5%)
No, I did not have what I needed, and I struggled	1 (12.5%)
Contraceptives used	
Yes	6 (75%)
No	2 (25%)

Five participants had lived in Ireland for more than 5 years. Many still struggled with English language proficiency, with only two women reporting that they could understand and speak English very well. Unsurprisingly, language barriers emerged as a major challenge. Although five participants reported needing a translator or interpreter at some point during their maternity care, only one participant reported being provided with an interpreter, while the others had to rely on family members or were left without any interpretation support.

During their first pregnancy in Ireland, four of the eight women were aware that maternity care was free, and only one participant was aware of the recommended antenatal appointments. Five women attended little or no antenatal appointments before their first delivery in Ireland. Despite this, almost all participants attended postnatal visits and received public health nurse visits.

Infant feeding practices varied, with four women using formula exclusively, while one woman breastfed exclusively. Financial struggles were evident, as four women required assistance with baby essentials, receiving support from hospitals, charities and family/friends. Contraceptive use was reported by six participants at least once during their lifetime.

## 5 Qualitative Findings

Focus group data was refined into an initial set of codes and then categorised into potential sub‐themes and main themes (Table 3). This structured approach enabled the capture of key patterns of Roma women's experiences, providing rich qualitative insights into the barriers, challenges and their engagement with healthcare services.

**Table 3:** Coding of thematic analysis.

Themes	Sub‐themes
Challenges of engaging with maternity services	Challenge of language and translator/interpreter availability
	Hospital expectations to bring their own translator/interpreter
	Reliance on family and friends
	Local healthcare and family constraints
Experiences with maternity healthcare services and its impact	Perception and experiences of discrimination
	Emotional distress due to communication barriers
	Mistrust due to uncertainty around treatment
	Preference for Irish maternity services in comparison to Romanian services
Cultural identity and maternity care needs and preferences	Preference for female health professionals for culturally sensitive communication
	Cultural identity and norms
	Mental health stigma
Poor health literacy and limited awareness of services	Limited awareness of maternity services
	Resource constraints
	Experience of using contraceptives

Roma women reported challenges at every level, from engaging with healthcare services to applying for social support and child benefits.

Four key themes emerged from the data analysis:

a. Challenges of engaging with maternity services.
b. Experiences with maternity healthcare services and their impact.
c. Cultural identity and maternity care needs and preferences.
d. Poor health literacy and limited awareness of services.

### 5.1 Challenges of Engaging With Maternity Services

Roma women experienced significant challenges when engaging with maternity services. Their interactions with the healthcare system were shaped by multiple barriers, including language difficulties, travel constraints and family responsibilities at home. Many women reported delays in accessing antenatal care or seeking healthcare for pregnancy complications.

#### 5.1.1 Challenge of Language and Translator/Interpreter Availability

A recurring challenge for Roma women was communicating with healthcare professionals due to limited English language proficiency. Many participants missed antenatal appointments because they were unable to understand the instructions provided or communicate their needs effectively due to the non‐availability of translators and interpreters, leaving them reluctant to attend hospital visits on their own.

‘I have six children, and I am expecting another baby. I am already 4 months pregnant, and didn't attend any appointments because I don't know how to explain anything in English and don't have anybody to bring to the appointments to interpret for me.’

(p4)

Past pregnancy experiences where no translator/interpreter was provided led them to assume they would not receive this support in future pregnancies, reinforcing their hesitancy to seek care.

Some Roma women faced significant barriers in accessing birth registration services for their newborns due to the absence of interpreters.

#### 5.1.2 Hospital Expectations to Bring Their Own Translator/Interpreter

Many women reported that hospital staff assumed Roma women would bring their own translator/interpreter, thus placing an additional burden on them. In many cases, women were left without any language assistance despite repeated requests.

‘I don't know how to explain my pain to them. The doctor told me, “Can you bring your interpreter yourself? Call someone.” But I don't know who to call.’

(p4)

‘I asked many times for an interpreter, but they told me to call someone who speaks English and bring them to the hospital to speak for me. No interpreter was provided, even though I was in the hospital for 2 days.’

(p8)

These challenges discouraged Roma women from attending regular check‐ups and hampered communication during hospitalisation.

#### 5.1.3 Reliance on Family and Friends

Due to low literacy rates, Roma women often rely on family members, neighbours or friends to read hospital letters, accompany them to appointments, and act as translators during hospital and public health nurse home visits (p4, p7).

‘They sent the letter by post with the appointment date. I have to take the letter to someone who could read it for me and tell me the date and time.’

(p4)

‘My family/friends call me to come to the Hospital to help because there was no interpreter. But sometimes, I couldn't go to the hospital, and I couldn't help.’ (p3)

#### 5.1.4 Local Healthcare and Family Constraints

Access to healthcare facilities was another major concern, particularly for women living in areas far from GP clinics or hospitals. ‘*I've been only to the hospital because my GP is far from where I live. So, I only see the doctor at the hospital’ (p3).*

Many Roma women felt that maternity services offered by GPs were limited, with the provision of services like urine testing, weight and blood pressure monitoring ‐ not meeting their expectations for pregnancy care. This led them to seek care directly at hospitals, contributing to delayed antenatal visits.

‘GPs don't have ultrasounds or scans or anything. They just check your blood and talk to you. I went a couple of times, but they don't really do anything at the GP.’

(p6)

Additionally, Roma women often prioritise family obligations over their healthcare and seek care at the final stages of pregnancy—‘*just at the time of delivery’, (p4)* or when urgent medical issues arose: ‘*when I had very sore pain and lost liquid’ (p2).*

‘With the gestational diabetes, I have three appointments a week and it's too much. I have two kids at home, and I can't attend all my appointments because there's barely anyone to mind them.’

(p6)

To improve healthcare access, participants suggested recruiting Roma women as interpreters and using communication channels, such as WhatsApp groups, to share information with pregnant Roma women in their own language.

### 5.2 Experiences With Maternity Healthcare Services and Their Impact

Roma women's experiences with maternity healthcare in Ireland reveal both negative and positive encounters, shaped largely by language barriers, perception and experiences of discrimination, and uncertainty around treatment.

#### 5.2.1 Perception and Experiences of Discrimination

Roma women reported experiencing discrimination from hospital security staff, making them feel unwelcome in healthcare settings. However, others reported positive interactions with doctors and nurses.

‘All the doctors and staff are very good and treat us same’ (p8) and ‘with the respect’ (p1). ‘The only problem at the hospital is the security. I feel like discriminated because the security person follows me and ask me questions, even though saw me talking to the secretary and in camera in front of him. I feel he's looking at me like he has no trust in me, and he may be thinking, that I'm going to make some problem.’

(p1)

#### 5.2.2 Emotional Distress Due to Communication Barriers

Due to poor communication, women felt ignored as they were unable to describe their symptoms clearly, leading to frustration and emotional distress.

‘I was explaining to them “I have pain, I have pain”—but the doctor and nurses didn't understand what kind of pain because I didn't know how to explain. He left me in the bed. He didn't think about me. After 30 min, I called them again. I was screaming, I was crying, but nobody cared’.

(p2)

The fear and stress were further amplified when medical procedures explained by healthcare practitioners were not understood by the individuals, leaving them confused and terrified.

‘It was very hard, very stressful, and difficult. I was in severe pain. They took me to another hospital for a big scan, but they didn't bring me a translator/interpreter. I was so afraid because they put me inside the tube, and I didn't understand what was happening’.

(p4)

#### 5.2.3 Mistrust due to Uncertainty Around Treatment

Most women were happy with the care provided at the hospital, and they believed *‘doctors and nurses were good and provided them with true and correct information and reassuranc*e*’ (p1, p3, p8*), gaining their trust.

However, in some cases, language barrier and lack of translator/interpreters resulted in delays in receiving medical care, as doctors refuse to proceed without an interpreter, leaving women in pain or untreated.

‘The doctor said to my relative (acting as interpreter on the phone), “If you are not coming, I'm not doing the injection.” He left me in pain and did nothing for an hour and a half until she arrived.’

(p8)

This uncertainty surrounding their treatment adds to their feeling of insecurity, especially when they cannot read consent forms or understand medical procedures.

‘I told her’ (p8), ‘Don't sign anything because you don't have an interpreter. You don't even know what the letter says. I was afraid, they might do a C‐section without her knowing.’

(p2)

‘When I went to the hospital for delivery, I didn't understand anything about what I had to do because I was on my own. The doctor did whatever he wanted with me. He gave injection on the back, and I didn't understand anything—what was this injection for? He didn't even explain to me and said—you don't understand. I told you to bring the interpreter, but you didn't’

(p4)

Following negative experiences, these women *‘chose not to complaint and remain silent’ (p1, p5)* as they were *‘unaware of the complaints process’ (p3, p8) and ‘feared negative consequences’* (p2).

#### 5.2.4 Preference for Irish Maternity Services in Comparison to Romanian

Most participants had strong preferences for Irish maternity services compared to Romania. They appreciated the respectful treatment and affordability of Irish maternity services in contrast to the Romanian system, where *‘if you pay the doctor money, the doctor will help you’ (p1, p8).*

‘In Ireland, doctor give a lot of respect in comparison to Romania’ (p5) and ‘makes me feel like it's in my own country’.

(p3)

Other reasons for preference of Irish healthcare were *‘shorter duration of hospital stay, provision of basic services like food for mother and baby, nappies in the hospitals and allowing husband to accompany during the appointments and delivery’ (p1, p8).*

### 5.3 Cultural Identity and Maternity Care Needs and Preferences

Roma women's cultural identity and traditional maternity practices significantly shape their experiences. Their beliefs, customs and social norms influence their engagement with maternity services.

#### 5.3.1 Preference for Female Health Professionals for Culturally Sensitive Communication

Roma women feel embarrassed and ashamed when discussing reproductive health issues with male doctors or male interpreters, resulting in reluctance to seek care or discuss health concerns. They strongly prefer female doctors for pregnancy‐related treatments, childbirth and gynaecological procedures, *due to cultural and religious beliefs (p1)*.

‘It's very important to have a female interpreter because a Roma woman won't explain everything to a man.’

(p1)

‘In my country, it is shameful to be assisted by a man when giving birth—even if he is my husband.’

(p2)

‘I feel more comfortable talking with a female doctor about my last menstruation or how I feel. I just don't feel well when I talk with men’. ‘I refused my appointment with a male doctor to insert Mirena coil as I only wanted a woman to insert it.’

(p3)

#### 5.3.2 Cultural Identity and Norms

Most Roma women strongly identify with their cultural heritage and take pride in their language, traditions and clothing. They feel a deep connection to their Roma identity.

‘It's visible from the way I dressed that I am from the Roma community. I don't have to explain it.’

(p1)

Sometimes hospital environments conflict with their cultural values and traditions, leading to discomfort, for example, the requirement to wear hospital gowns.

‘I couldn't wear the short dresses they gave me in the hospital. I was so shy.’

(p6)

Roma women adhere to traditional maternity customs passed down through generations, which sometimes differ from modern medical practices, for example, ‘*wrapping newborns tightly in blankets for 6 weeks to strengthen their bones* and *not buying baby clothes before birth due to superstitions about bad luck’ (p1, p2)*, and ‘*relying on knowledge passed down from their mothers and grandmothers’ (p6)*.

#### 5.3.3 Mental Health Stigma

Seeking mental health support is stigmatised within Roma communities. Women are reluctant to attend specialists or therapy, fearing judgement from others in their community.

‘Roma people don't allow going to speak with a specialist or to the hospital for mental health. In our community, this is a shame. If someone hears that you went to check, they think something is wrong with you.’

(p1)

While many feel overwhelmed by their responsibilities, they do not openly acknowledge mental health difficulties. Instead, they internalise these, choosing to stay silent whilst focusing on household and childcare responsibilities to avoid dwelling on emotional struggles.

‘Depression is not something people talk about.’ (p1)

‘Roma women's lives are busy every single day. There's no time to feel bad. Even if I have a problem, I still have to cook and clean, even if I'm sick.’

(p1)

### 5.4 Poor Health Literacy and Limited Awareness of Services

Roma women have limited awareness of healthcare services and social supports available, leaving them without essential supplies during pregnancy and postpartum. Additionally, decisions about birth control are heavily influenced by personal experiences and informal discussions with family/friends.

#### 5.4.1 Limited Awareness of Maternity Services

Most were unaware of antenatal classes or additional maternity services that could improve their pregnancy experience. They often rely on word‐of‐mouth recommendations when choosing a maternity hospital, limiting their exposure to a broader range of healthcare choices available.

‘I never heard of other hospitals. I only know about this hospital from my sister‐in‐law and friends. They always say it's the best hospital.’

(p3)

Some women indicated that, due to their low literacy levels, they cannot read the postnatal care information pamphlet provided to them.

#### 5.4.2 Resource Constraints

Many Roma women felt unable to explain their financial difficulties to hospital staff or social workers due to their limited English, and they struggled to access social support and child benefits due to a lack of knowledge about application processes and criteria.

‘It was very difficult because I had no social payments. I didn't know how to get a buggy or baby clothes. There was no one to explain how to do the letters, how to apply for social support, and I didn't know how to seek help because there was no interpreter.’

(p4)

#### 5.4.3 Experience of Using Contraceptives

Their decisions about contraception usage were often based on fear of side effects, personal experiences, and advice from family and friends rather than healthcare staff, leading to misconceptions.

‘I didn't use contraception because I heard it can change your body—some get fat, or too skinny. My sister‐in‐law got an injection in the arm, and after that, she became bigger and bigger.’

(p1)

Some participants reported that physical side effects made them uncomfortable and led them to stop using birth control entirely, while some associated contraceptives with infertility issues.

‘That injection didn't feel right for me. It made me very skinny, gave me headaches, and I couldn't get pregnant for 2 years.’

(p8)

‘I used the coil for 4 years, but I never felt okay. I had headaches, felt tired all the time, and my periods never stopped. Eventually, I went to the hospital and asked them to take it out.’

(p2)

These experiences highlight the need for culturally sensitive contraceptive education, where healthcare providers can address concerns, provide accurate information, and ensure women make informed choices about family planning.

## 6 Discussion

In this research study, eight Roma women shared their experiences of maternity and neonatal services in Ireland. Most had early pregnancies before the age of 19 years, large families, and limited English proficiency, with many requiring support. While awareness of free maternity care and attendance at antenatal services was low during their first pregnancies, postnatal care was more consistently accessed. Financial hardship, infant feeding practices and contraceptive use varied among participants. Participants faced challenges in accessing maternity and social services due to low health literacy, previous experience and cultural preferences.

The study findings align with international literature, which highlights that Roma women are 23 times more likely to give birth as minors (< 18 years) compared to their non‐Roma counterparts—a factor associated with increased risks of preterm birth, low birth weight and higher maternal mortality rates [8, 26]. These outcomes are further compounded by systemic disparities in maternity care experienced by marginalised ethnic communities, shaped by intersecting factors such as ethnicity, race, religion, socio‐economic status, age, marital status and sexual orientation [2, 8, 13, 27]. For many Roma women, pregnancy marks their first sustained engagement with the healthcare system, making these early interactions critical for building trust and influencing future health‐seeking behaviours. Failure to provide this may lead to long‐term disengagement from health services and persistent health inequities [28].

Roma women reported multiple barriers to accessing care, including limited awareness of services [10, 29], transport barriers, financial constraints [29, 30], and relying on family/friends due to a lack of interpreters/translators. Studies show that language barriers prevent effective communication with healthcare professionals, leading to missed antenatal appointments and delays in care [2, 8, 13]. Reliance on family members to act as translators compromises the quality of information exchanged, reducing trust in healthcare providers and raising concerns regarding medical confidentiality when discussing sensitive matters [2, 6, 10].

Some described feeling ignored or neglected during labour because they could not adequately explain their symptoms due to the language barrier. Others encountered traumatic experiences of refusal of care or undergoing treatment without a clear understanding of what was happening, leaving them in distress for prolonged periods. Previous research also highlights that the lack of clear communication heightens the feelings of fear and vulnerability among Roma women [29, 31].

Roma women reported feeling unwelcome due to instances of discriminatory and distrustful behaviour by some hospital staff. This reflects wider European antigypsyism [32, 33] behaviour where Roma women have reported being discriminated [34], physically and verbally abused [35], not treated with respect [10], being ignored or abandoned in hospitals [31, 36], denied treatment [31, 37], or receiving lower‐quality care due to racial prejudice [26, 28]. However, Roma women in this study also expressed their overall satisfaction with the treatment provided by doctors and nurses in comparison to Romania, indicating that while systemic barriers persist, most healthcare professionals in Ireland provided compassionate and respectful care [6, 16, 29].

Cultural identity and traditional practices significantly shape the preferences and experiences of Roma women. They expressed discomfort with male doctors assisting during childbirth, citing cultural and religious reasons, which is consistent with literature citing that Roma women prioritise gender‐matched healthcare providers for reproductive and maternity care [13]. Research suggests that healthcare systems that fail to accommodate cultural beliefs risk further alienating Roma women from formal maternity care [10, 13].

Many of the women reported prioritising family responsibilities over personal well‐being, leading to emotional exhaustion, but dismissed their emotional well‐being, stating that they have no time to focus on their emotions. This aligns with other research studies indicating that mental health is a low priority within Roma communities and is often overlooked due to cultural stigmatisation, with many individuals experiencing frequent mental distress but not seeking support services [13]. The reluctance to seek professional assistance highlights the need for community‐based interventions that normalise mental health discussions and provide culturally appropriate support mechanisms.

Limited health literacy was a common challenge, with many Roma women in this study relying on word‐of‐mouth recommendations. Available literature depicts that this limited awareness of available services, including antenatal classes and supports, social and child benefits, and contraceptives [38] prevent Roma women from fully utilising maternity services [17, 18].

## 7 Implications and Recommendations

Findings of this study emphasise the urgent need for systemic interventions to improve maternity care for Roma women. Key recommendations include:

- Addressing systemic issues such as the lack of access to adequate primary care [12, 20, 21] through targeted practices such as clear and easy process of registration with GP, reducing waiting time, delivering care close to accommodation and provision of more services at GP level.
- Strengthening community outreach programmes to raise awareness of maternity services, social entitlements and contraceptive options [13].
- Ensuring availability of culturally appropriate professional translator/interpreter services in all maternity units to improve communication and safety and reduce medical uncertainties [13].
- Addressing the inclusion of an ethnic identifier in healthcare data collection to monitor disparities and evaluate and improve health outcomes [10, 39].
- Addressing systemic discrimination and institutional racism in health settings through clear and accessible complaints processes for patients with low literacy levels, and broader efforts to tackle systemic discrimination by providing training to recognise antigypsyism, its impact, targeted policy reform, and the employment of minority ethnic groups at all levels [33].
- Providing anti‐racism and discrimination training for all staff working in healthcare facilities to raise awareness [10].
- Providing an opportunity to discuss mental health issues during regular antenatal appointments in a culturally appropriate and sensitive manner [13].
- Fund community‐based healthcare initiatives led by organisations that are trusted by and embedded within the communities they serve.

## 8 Strengths and Limitations

Integrating healthcare education into the FGDs provided context for the participants to relate their experiences, as well as simultaneously imparting knowledge to these women. Structuring sessions around the pregnancy journey provided structure and focus. Partnering with Cairde greatly facilitated engagement with the Roma community, overcoming language and literacy challenges and improving data quality. The online format, supported by the provision of necessary digital devices, further enhanced accessibility and improved participation.

While offering valuable insights, this study is limited by its small sample size of eight participants, which may not reflect the full diversity of Roma women's experiences. Recruiting through Cairde may have introduced selection bias, excluding less‐connected individuals. Self‐reported data from focus groups and online questionnaires may be affected by recall and social desirability bias. Language barriers and informal interpretation methods may have led to miscommunication. Though the online format enabled participation by reducing barriers like childcare and travel, it may have limited the depth of interaction. Future research should involve a larger, more diverse sample to strengthen findings.

## 9 Conclusion

This study highlights the significant barriers Roma women face in accessing maternity care in Ireland, including language difficulties, financial hardship, cultural factors and discrimination. Limited English proficiency, lack of interpreters and unfamiliarity with antenatal services often lead to delayed or inadequate care, increasing health risks. Systemic issues—such as discriminatory treatment by hospital staff, lack of culturally competent care, and poor awareness of available services—further compound these challenges.

Despite these challenges, many Roma women appreciated the respectful treatment provided by Irish healthcare staff. However, low health literacy, reliance on informal information sources, and stigma around mental health underscore the need for targeted interventions.

Addressing these disparities requires professional, culturally appropriate interpretation services, culturally tailored health education through Roma peer networks, improved access to services, and policy reforms to counter institutional discrimination. Future research should include comprehensive health equity assessments, considering broader social determinants like housing, education, employment and health behaviours. Prioritising culturally responsive care and building trust can help ensure equitable, high‐quality maternity care for Roma women.

## Author Contributions

**Jaspreet Kaur Dullat:** conceptualisation, methodology, investigation, formal analysis, visualisation, writing – original draft preparation, writing – review and editing. **Thérèse McDonnell:** conceptualisation, project administration, methodology, investigation, formal analysis, visualisation, validation, writing – review and editing. **Louise Hendrick:** resources, validation, methodology, writing – review and editing. **Léan McMahon:** writing – review and editing. **Gemma Moore:** validation, writing – review and editing. **Marianna Prontera:** project administration, resources, methodology, validation, writing – review and editing. **Emily Murphy:** resources, project administration, methodology, validation, writing – review and editing. **Carmen Nae:** resources, project administration, validation. **Danut Nae:** resources, project administration. **Eilish McAuliffe:** conceptualisation, formal analysis, funding acquisition, writing – review and editing.

## Ethics Statement

This study has received ethical approval from UCD Human Research Ethics Committee‐ Sciences (Reference Number: LS‐LR‐23‐184‐McAuliffe) and the Research Ethics Committee Midlands Area and Corporate (Regional Health Area B) of the Health Service Executive (Study: Evaluation of development and proposed implementation of the Quality and Safety Signals Proof of Concept in Maternity and Neonatal Services, Ref: RRECB1123 L.H.).

## Conflicts of Interest

The authors declare no conflicts of interest.

## Supporting information

Findings_Appendix_anonymized.

## Data Availability

The anonymised data that support the findings of this study is available upon reasonable request from the corresponding author.
