# Supplementary material for: Understanding a Minority Group's (Roma) Experiences of Access and Quality in Maternity Services
Source: Health Expect. 2025 Aug 18;28(4):e70389. doi: 10.1111/hex.70389 (PMC12361635; doi:10.1111/hex.70389)
Supplement: Supplementary file 1 — Findings_Appendix_anonymized. [file HEX-28-e70389-s001.docx]

|  |  | |
| --- | --- | --- |
| **APPENDIX A** | **FOCUS GROUP QUESTIONS** | |
| **APPENDIX B** | **PARTICIPANT INFORMATION LEAFLET** | |
| **APPENDIX C** | **CONSENT FORM** | |
| **APPENDIX D** | **QUESTIONNAIRE** | |
|  | |  |
|  | |  |

**APPENDIX A: FOCUS GROUP QUESTIONS GUIDE**

| **WORKSHOP 1 – ACCESSING ANTENATAL CARE & BARRIERS; LANGUAGE ISSUES** | |
| --- | --- |
| **Accessing healthcare during pregnancy** | - A list of all the visits you should have with a GP and the hospital was presented. For those of you who have had a baby in Ireland, were the things explained by similar to your own experience? Did you have that many visits or some visits before you gave birth? - Did you attend your antenatal appointments with your GP or the hospital? - Do you trust the healthcare workers (the doctors and the nurses) you met in the hospital? - Did you attend antenatal classes, or were you offered antenatal classes by healthcare workers? - Did anybody have a conversation about Antenatal classes with a nurse or a doctor at your early visits to the hospital? |
| **Barriers to access** | - Has there ever been a time during your pregnancy, when you worried about something about you or the baby, but you were not comfortable seeking healthcare? - When you were pregnant were there times when you didn't want to go to the hospital?  Maybe you had a bad experience, felt you were being invited to too many appointments, or had other reasons you didn't attend your appointments. - Was the only reason you didn't have the Down’ Syndrome test because of the cost and this test wasn’t covered by a medical card? - Did you feel that you don’t need antenatal classes because of your experience helping or parenting your siblings? |
| **Language (at any stage during pregnancy/birth)** | - Have you ever experienced situations where you asked a translator, but there wasn’t any available? - Has anybody ever tried to explain to a nurse or a doctor that not having an interpreter is a problem but having a male interpreter is also an issue?   Have you told the hospital staff about your culture, and how you feel comfortable having a female doctor at the time of delivery instead of men? |

| **WORKSHOP 2: HAVING YOUR BABY** | |
| --- | --- |
| **Accessing healthcare during pregnancy and labour** | - What was your healthcare experience while you were pregnant and, in the hospital, having your baby? - As you said, healthcare in Romania is very different from Ireland, how does your experience in Ireland compare to your home country? - Did your husband accompany you to the hospital for appointments or having the baby, or do you prefer to go on your own or with a female friend? - How many weeks pregnant were you when you attended a doctor/hospital in Ireland as high-risk? - After giving birth, did you stay in the hospital for the recommended length of time? |
| **Barriers to access** | - What were the reasons for not attending/ missing appointments? - If your GP was near you, would you prefer going to your own doctor than to the hospital? |
| **Respect and Trust** | - When you went to the hospital near the end of your pregnancy, how did you feel about the staff for you? Were they angry, or were they kind and supportive? - Did the staff explain the procedure or check-up they are going to perform? Did they seek your permission? |
| **Epidural** | - Did you understand what was happening while administering Epidural or was that a challenge? |
| **Cultural Identity** | - When you were in the hospital, did you always feel comfortable identifying as Roma? - Was it important to you that the staff knew you were Roma, and why or why not? |

| **WORKSHOPS 3 & 4:  POSTNATAL CARE, MENTAL HEALTH, AND FOLLOW-UP QUESTIONS FROM PRIOR WORKSHOPS**  (2 breakout sessions of 2 focus groups: workshop 3 and 4) | |
| --- | --- |
| **Postnatal Care** | - When you had your first baby did you feel you knew how to take care of yourself after leaving the hospital? Did the people around you, such as family or friends, help you with this? - Did someone from your family/ hospital help you, if you had any problem? - Did anyone not have help after leaving the hospital? - When you left the hospital, did you have all the things that you needed to take care of the baby? If not, who helped you figure out what you needed and where to get it? - Did the hospital staff provide you with information about postnatal care, or did you already know about how to take care of yourself and your baby? - Did the hospital staff provide any classes or information to you after the delivery regarding postnatal care and newborn care? - Did you get the support from hospital staff or the Public Health Nurse (PHN) in Ireland after your delivery? - Did the Public Health Nurse (PHN) visit your home to check on you and your baby? Did you feel the visit was enough? - Did the PHN explain your and your baby's condition? Were you able to understand the information provided by PHN? - Did you see your GP after giving birth for follow-up care? - After your delivery, did you attend your and your baby’s postnatal check-ups? - Why is Rotunda a hospital of choice? |
| **Breastfeeding** | - Did you breastfeed your baby? - What is your preferred method of feeding your baby- breastfeeding or bottle feeding? - Did you know about breast pump? - While feeding your baby, did you experience any problems, such as the baby not taking enough feed? How did you address these challenges? |
| **Contraception** | - After your previous deliveries, have you used contraceptives? - Did you know about the options available? Who gave you that information? |
| **Mental Health** | - How did you find you were in terms of your mental health? - Did you ever feel depressed or sad after the birth of your baby? And if so, what did you do? - Did you talk to someone about your feelings, such as a family member, friend, or healthcare professional? |
| **Cultural Identity** | - When you go to the hospital, do you feel proud to identify yourself as Roma, or are there any occasions you try to hide your identity? - Did you encounter any prejudice or unfair treatment in the hospital because you are from the Roma community? - Did you feel that the hospital staff respected you and tried to do things according to your choices? - Do you feel comfortable identifying as a Roma woman in healthcare settings? |
| **Respect and Trust** | - Did you have any problem with the staff or doctors during your scans, or when you were in the hospital for delivery? |
| **Making a Complaint** | - If you felt uncomfortable, did you try to make a complaint to someone or did you try to talk to someone that I am feeling uncomfortable? - When you had a bad experience such as not having an interpreter, did you ever consider making a complaint? - Has anyone ever complained about not getting the support they needed, such as an interpreter? - In the future, if you feel you face any problems, or you have some complaints. Would you like to make a complaint, or you would like to be quiet? |

| **WORKSHOP 4: GROUP SESSION**: **DESIGNING A MATERNITY SERVICE TO MEET THE NEEDS OF ROMA WOMEN AND THEIR BABIES**  (1 focus group in workshop 4) | |
| --- | --- |
| **Interpreter** | - When you had your first visit, to the hospital, was that an appointment? Did the hospital know you were coming? If you had an issue with English, did they provide you with an interpreter? - Did you request an interpreter, or did they tell you to bring your interpreter? - Did anyone ever ask for an interpreter during your visits? - Have the doctors or nurses tried to use tools like Google Translate to help with communication? |
| **Antenatal Classes** | - If we want to do antenatal classes for young, new mothers or young mothers, young girls, and women who are pregnant, What's the best way to do that- online or going into their communities? - Are there any parenting groups or organizations that you are involved within your community? Where do Roma families meet in your community? - Would you like to have the opportunity to meet for the same reason? Do you think it's a good idea for new mothers to meet in person and bring their babies together, say, once a week or in a community setting? |
| **Changes In Perspective** | - What advice would you give your daughter now about, starting a family? For instance, what age do you think is a good age to start your family? |

**APPENDIX B: PARTICIPANT INFORMATION LEAFLET**

**Study Title: Developing a national suite of measures of the quality and safety of Maternity and Neonatal services for inclusion in a national surveillance system (Quality and Safety Signals)**

You are kindly invited to take part in this research project to develop a national suite of measures of the quality and safety of Maternity and Neonatal services for inclusion in a national surveillance system (Quality and Safety Signals).

Before you decide whether or not to participate, we would like you to understand why the research is being done and what it would involve for you. Please take your time to read this information. You can ask us if there is anything that is not clear, or if you would like further information or if you have any questions.

**Information about this study**

**What is this research and why is the research being done?**

This research project aims to develop a national suite of measures of the quality and safety of Maternity and Neonatal services for inclusion in a national surveillance system (Quality and Safety Signals). This system will integrate, analyze and display quality, safety and operational data from multiple different datasets in one place to maximize insights on the quality and safety of Maternity & Neonatal services.

This research project will initially use information from international literature and consultation with a Clinical Advisory Group (CAG) to identify measures for inclusion in a suite of measures to monitor the quality and safety of Maternity and Neonatal healthcare services. Identified measures will then be further assessed by a panel of service users to ensure the relevance, completeness, and importance of identified measures.

**Why have I been asked to take part?**

The involvement of service users and the wider public in designing, planning and co-producing healthcare brings an important perspective and highlights the issues that are relevant to service users. Therefore, involving service users in the selection and review of relevant measures of quality and safety is an important aspect of the co-design process. You have been identified as a service user who can provide feedback on the importance and completeness of measures identified in the initial phase of this study.

**Do I have to take part?**

Participation in this study is entirely voluntary. It is up to you to decide whether or not you would like to take part. If you agree to take part, we will ask you to sign a consent form. You are free to refuse to take part, or to withdraw at any point, without giving a reason and without any adverse effects as a result.

**What will happen if I agree to take part in this research study?**

If you agree to take part, the research team will ask you to sign a consent form (in person or virtually). You will be given a chance to ask any questions you may have before agreeing to take part.

By opting into the study, you will be asked to take part in 3 rounds of co-design online workshops, lasting for 45-60 minutes, and 2 online surveys, lasting for 10-15 minutes respectively which will focus on your views on the importance and completeness of shortlisted measures. The workshops will be conducted using an online meeting platform at the date and time most convenient for you. The researcher will introduce themselves and answer any questions you may have about the study. With your permission, we will audio-record the workshops. This recording ensures the Research Team captures all aspects of the workshop discussion. All the information collected will be redacted and pseudonymized to protect the identity of participating staff and your views will be kept confidential.

**What are the possible benefits of taking part?**

While there may be no immediate direct benefit to you of taking part, the results from this study will ensure the service user’s perspective on the importance and completeness of the measures shortlisted is taken into account when agreeing the final suite of measures for inclusion in a Quality and Safety surveillance system of Maternity and Neonatal services.

**What are the possible risks of taking part?**

We do not envisage any harm to participants due to their participation in the research. Please remember that you are free to end your participation in the study at any time, and this will not affect your rights in any way.

Should any aspect of the discussion in the virtual workshops or survey questionnaires upset you such that you feel you need support, please contact your General Practitioner or other relevant health care professional.

**Will I receive any expenses or payments?**

We will not provide any additional payments for your participation except travel expenses that were incurred by getting involved in this research.

**What will happen if I change my mind about taking part?**

If you agree to participate in the study, but later decide that you no longer wish to take part in any subsequent workshops and/or surveys, please contact a member of research team quoting your unique identification number that is printed on the consent form.

**Will my taking part in the study be kept confidential?**

The audio-recordings of the workshops will be transcribed and pseudonymized, and personal information that could potentially identify you will be removed. Personal identifiers will also be removed from the survey responses on completion. Your name or any other information that could potentially identify you will not appear in reports, publications or presentations arising from the research. We may want to use direct quotes from participants who take part in the interviews, but quotes will not be attributed to individuals.

Data will be managed securely. All notes, transcripts and results will be stored electronically and will be securely stored on password protected cloud storage, with access restricted to named members of the Research Team through their designated login IDs. The electronic versions of the data collected will be held for five years then securely destroyed.

**What will happen to the results of the study?**

The results of this work will provide an evaluation of the indicators/measures derived during the project and act as a guide for subsequent implementation of a Quality and Safety surveillance system (QS Signals). We intend to publish the findings in reports and scientific journals and to present them at scientific meetings and international conferences. Any information that could potentially identify you will not be included in any report or publication.

**How will I find out what happens with this project?**

If you would like to receive a summary of the findings related to this phase of the research, you can notify the researcher that you would like to provide your contact details to be contacted for this purpose.

**What happens next?**

If you are happy to proceed, we will explain the study and answer any questions you may have. If you are interested in taking part in interviews, we will ask you about dates and times that would be convenient for you to participate virtually.

If you have any concerns about the study, or would like more information, please contact us.

**Thank you for taking the time to read this information**

**APPENDIX C: CONSENT FORM**

**Service User Panel**

Developing a national suite of measures of the Quality and Safety of Maternity and Neonatal Services for inclusion in a national surveillance system (Quality and Safety Signals)

After reading the information material, if you agree to take part in the study, please conﬁrm your consent by selecting all the boxes below and typing in your name at the bottom of this form.

- I have read the study-related information and understood it. I had time to think about it and ask questions.
- I understand that I do not have to take part in this study, and I can leave whenever I want, without giving any reason.
- I agree to take part in 3 online workshops and 1 online survey.
- I understand that the workshops will be audio-recorded so what I say can be written down by the Researchers, and that the recordings will then be deleted.
- I understand that the workshop ﬁndings or results might be published in research papers, but my name will not be disclosed anywhere.
- I understand that other than my consent form, information recorded during the study will not mention my name or other information that might identify me
- I agree that this consent form, which includes my name, will be saved on a secure online drive by the research team for ten years and then destroyed.
- I agree to take part in this study.

Your Name:

________

**APPENDIX D: QUESTIONNAIRE**

**(Anonymous)**

Thank you for joining our workshops. We would like to ask you some questions so we can understand your story when you were pregnant and had your babies.

Your answers are important to us, but if you are not comfortable answering any of these questions, you do not have to answer.

We ask your name so we can keep track of who has completed the survey, but all names will be removed once the survey finishes.

We will keep all your answers private. Your answers will be grouped together and will help people who work in the maternity services to improve the experience of Roma women in the future.

**PART 1 - BACKGROUND**

***About You***

**What age are you?** type in number

**How many years have you lived in Ireland in total?** type in number

**How many children do you have?** type in number

**How many were born in Ireland?** type in number

**What age were you when you were first pregnant, including miscarriage?** type in number

**What age is your youngest child now?** type in number

**Are you pregnant now?** Yes/No/rather not say

**Do you live with a husband/partner?** Yes/No/rather not say

**Where were you born:** Ireland, Romania, Poland, Slovakia, Other (box)

**What languages do you speak {tick all]:** Roma, Romanian, Czech, Slovak, Polish, English, Other (box).

**Can you read a story to your child in any of these languages?**

I cannot read, Roma, Romanian, Czech, Slovak, Polish, English, Other (box).

**How well do you speak English?**

Not at all,

not well,

well,

very well

**Can you read a story in English to your child?** Yes/No

***Housing***

**What kind of accommodation do you live in currently?**

Social Housing (rental), Private Rental, Emergency Accommodation, Other (box)

**How many adults and children live in your accommodation?**

Adults (type in number) Children (type in number)

**How many bedrooms are there in your accommodation?** (type in number)

**Do you feel there is enough space in your accommodation for everyone?** Yes/no/rather not say

**If no, can you explain [box]**

**How would you rate the standard of your accommodation?**

very bad, bad, ok, good, very good.

**Can you explain your rating:** Comment box

**Since your first child was born, have you experienced homelessness?**

Yes/No/Rather not say

**If yes, did you experience homelessness in Ireland?**

Yes/No/Rather not say

***PPS Number/Medical Card***

**During your first pregnancy in Ireland, did you have a PPS number**? Yes/No

**If no:**

- **was it because of the Habitual Residence Condition?** Yes/No/Don’t know
- **did you have a problem getting medical care at the hospital when you were pregnant?** Yes/No
- **did you have a problem getting medical care with a family doctor/GP when you were pregnant?** Yes/No/Did not try
- **did you use the SafetyNet Service** Yes/No
- **did you get medical help anywhere else?** Comment box
- **Do you have a PPS number now?** Yes/No

**You do not need to have a medical card to get free maternity care in Ireland. The medical card does give you access to other medical care at no charge. The GP visit card gives you access to a family doctor at no charge.**

**Did you have a medical card (Yes/No) or GP visit card (Yes/No)** **when you were first pregnant in Ireland?** Yes/No

**If no, do you have a medical card (Yes/No) or GP visit card (Yes/No) now?**

**If no, have you applied for a medical card or GP visit card?**

- Yes: (how many months since you applied) a medical card or GP visit card (tick box)
- No: I did not know about the medical or GP visit card;
- No: I don’t need/want one
- No other reason (comment)

***Having enough money to provide for your baby and family***

**After your first baby was born in Ireland, did you have what you needed to care for them: cot, nappies, buggy, bottles, formula (if not breastfed), clean water, clothes?**

- Yes, I didn’t need any help
- Yes, the hospital helped.
- Yes, I was provided with help but not from the hospital (e.g. Charity)
- Yes, friends and/or family helped.
- No, I did not have what I needed but I managed. No, I did not have what I needed and I struggled

**In general, how difficult is it for you and your family to manage with the money you have?**

- we can manage with money to spare/left over,
- we have enough to manage with no money left over,
- we might not have enough some weeks
- we do not have enough money every week
- rather not say

***Your Identity as a Roma Woman***

**When accessing healthcare, are you happy to tell healthcare staff that you are Roma?**

- Yes, I tell them I am Roma if they ask
- No, I am afraid the staff may not treat me well
- No, I don’t feel the staff can relate to me
- No, I don’t think it’s important.
- No, cannot communicate as don’t speak English/lack of interpreter
- No, I’d rather not say why
- No, Other reason (comment box)

**PART 2 - HEALTHCARE**

**When you were first pregnant in Ireland, did you know that maternity care is free if you are ordinarily resident, even if you don’t have a medical card?** Yes/No

***Need for an Interpreter for visits/appointments***

**Did you ever need an interpreter at the hospital?** Yes/No

***If YES, 2 columns: 1. For appointments while pregnant 2. when in hospital (e.g when giving birth)***

**If yes, did you ever request an interpreter:**

- Yes, before I attended.
- Yes, when I got there.
- Did not ask.

**If you requested an interpreter, did you ask for a:**

- female interpreter yes/no
- Roma interpreter yes/no

**In general was an interpreter provided by the hospital?**

- Always
- Sometimes
- Never
- I was told to bring someone who could interpret

**In general, was the interpreter provided:**

- Roma: Yes/No
- Female: Yes/No
- No interpreter
- Interpreter did not speak my language

**If the hospital provided an interpreter, was this [tick all]:**

- On the phone
- In person
- Internet (e.g. Zoom)

**If the hospital provided an interpreter, was it for as long as you needed them**? Yes/No

**If there was no interpreter available, who did you get to interpret [tick all relevant]**

- No one as no one was available
- No one as I don’t want to share my medical information
- One of my children aged under 18 years
- One of my children aged 18 years or over
- A family member or friend (female)
- A family member or friend (male)

**Overall, how much of what you were told did you understand**

**without an interpreter, with family/friend interpreting, with a professional interpreter?** [A column for each of the 3 options]

- I understood nothing;
- I understood a little but I did not fully understand the medical terms and information
- I understood a lot but not everything;
- I understood everything

**Making a Complaint**

**If you had a bad experience at the hospital, would you make a complaint?** Yes/No

**If no, why not (can tick all):**

- Did not know where and how to complain (had no help)
- I don’t feel comfortable complaining, ever
- I don’t feel it would change anything
- I am afraid the staff will treat me badly if I complain
- My English is not good enough
- Other reason (comment box)

**ANTENATAL**

**You were told you about all the appointments you should attend when you are pregnant. Did you know that you should attend these appointments?**

- Yes
- No
- I knew about some of these appointments but not all

**When you had your first baby in Ireland, did you attend your GP and/or hospital for pregnancy check-ups/appointments before the birth of your baby in Ireland?**

- Yes, all or most of my appointments
- Yes, one or two.
- No

**If you did not attend all or most appointments for your first baby born in Ireland, why not?**

- Did not need to as I was not sick
- I did not think there was any issue with the baby
- Didn’t understand as did not speak English/didn’t know about interpreters
- Did not live in Ireland during the pregnancy
- Concern about how I would be treated
- Didn’t know where to go.
- I thought it would cost too much money to see the doctor
- It cost too much money to get there (bus/train)
- Doctor/hospital was too far away
- Other (Box)

**If you had more than one baby born in Ireland, did you attend more or less appointments before the baby was born during these later pregnancies?**

- Only had one baby in Ireland
- The same number of visits as for first baby in Ireland
- More, because I understood the system better – accessing interpreters etc.
- More because I could understand more (English)
- More, because I trusted the system more
- More, for medical reasons
- More, I just felt I should
- Less, because I did not understand English
- Less, I felt I didn’t need the appointments
- Less, it was too difficult with other children to get to these appointments
- Less, because I had a negative experience/trusted the system less

**Did you attend your family doctor/GP when you were pregnant for antenatal appointments/ check-ups?**

- Yes, just once
- Yes, a few times
- No, I don’t have a GP
- No, my GP is too far away
- No, I prefer the hospital
- No, I had no appointments with my GP

**Did you have vaccinations during your last pregnancy (whooping cough, flu, covid)?** Yes/No

**If no, why not:**

- did not know about these vaccinations
- did not know it was important
- do not believe/trust in vaccinations
- thought they might be bad for my baby
- Other reason (box)

**IN HOSPITAL: LABOUR/BIRTH & HOSPITAL STAYS WHILE PREGNANT**

**If you had an epidural (injection in your back to stop you feeling pain) or other pain medication, did you understand what was happening and did you agree to this (consent)?** Yes I understood, no I did not understand

Yes I agreed, not I did not agree, I don’t know if I agreed

**Did you want an epidural (injection in your back to stop you feeling pain) or other pain medication, but could not because you could not communicate with the doctor (consent)?**

Yes/No/Don’t know

**Did you feel comfortable in the hospital (on the ward, with your bed, feeding baby, surroundings, noise)**

- Yes
- No
- If no, why not [text box]

**Overall, how happy were you with the medical care you received when you were in the hospital?**

Very unhappy; Unhappy; it was ok; happy; very happy

**Overall, how happy were you with how the doctors, nurses, and other staff in the hospital treated you when in hospital (respect, kindness)?**

Very unhappy; Unhappy; it was ok; happy; very happy

**AFTER YOU HAD YOUR BABY AND LEFT HOSPITAL**

**Thinking about your last pregnancy in Ireland, did you receive a visit at your home by the nurse when you and your baby left hospital?** Yes/No

**If yes, how many visits:** [number]

**Thinking about your last pregnancy in Ireland, did you attend** **your GP or the hospital for a check-up 6 weeks after your baby was born to check on how you and your baby were doing?** Yes/No

If no, why not? Comment box

**Did your last baby born in Ireland who is over 13 months old have their vaccinations at the following ages:**

2 months; 4 months; 6 months; 12 months; 13 months (should tick all boxes); none

**If not, why not? Freetext (or I didn’t know they needed them; I didn’t feel they needed them…)**

**After you had you baby, if you were worried about your health or the health of your baby, did you have a doctor, nurse or clinic you could attend?** Yes/No

After the baby follow-up appointments at 2 and 6 weeks, was your baby registered with a GP as part of the free GP scheme? Yes/No

**When your babies were very small (< 3 months) how did you feed them?**

- Only breastfed
- Only bottle fed
- Both breastfed and bottle fed

**When your babies were small (< 6 months) what milk did you use in bottle feeding**?

Formula, cow’s milk, other milk [text box]

**For how long did you breastfeed your baby?**

- I did not breastfeed
- Only the first few days
- 1 month
- 1-3 months
- 3-6 months
- 6-12 months
- Up to 2 years
- Longer than 2 years.

**If you did not breastfeed, why was this**: [comment box]

**What type of nappies do/did you use for your babies?**

cotton nappies (reusable); disposable nappies (single use); a mix of both

**Did you receive advice on contraception after the birth**: Yes/no

**Where did you get this advice? [tick all]** Hospital/Family doctor/public health nurse/ friends & family

**Have you ever used contraceptives?**: Yes/no

**PART 3 – FEEDBACK ON THE WORKSHOPS**

**Do you think the workshops conducted increased your understanding of: (Likert scale – 1 – 5)**

how to care for yourself while pregnant (workshop 1)

having a baby (workshop 2)

caring for you and your baby after the birth (workshop 3)

**Would you advise other women from the Roma Community to participate in classes or workshops like these?** Yes/No.

**Do you feel having access to classes like these would encourage Roma women to access care while pregnant?** Yes/No.

Would you like to comment:

**Would you change anything about how the workshops were conducted?** No/Yes.

If yes, what (comment box)

Thank you for completing this survey.
